# The Anxiolytic-like Properties of a Tryptic Hydrolysate of Bovine α_s1_ Casein Containing α-Casozepine Rely on GABA_A_ Receptor Benzodiazepine Binding Sites but Not the Vagus Nerve

**DOI:** 10.3390/nu14112212

**Published:** 2022-05-26

**Authors:** Simon Benoit, Catherine Chaumontet, Nicolas Violle, Audrey Boulier, Zeeshan Hafeez, Céline Cakir-Kiefer, Daniel Tomé, Jessica Schwarz, Laurent Miclo

**Affiliations:** 1 Université de Lorraine, INRAE, URAFPA, F-54000 Nancy, France; sim.bnoit@gmail.com (S.B.); celine.cakir-kiefer@univ-lorraine.fr (C.C.-K.); 2 UMR PNCA, AgroParisTech, INRAE, Université Paris-Saclay, F-75231 Paris, France; catherine.chaumontet@agroparistech.fr (C.C.); daniel.tome@agroparistech.fr (D.T.); 3 ETAP-Lab, F-54500 Vandœuvre-lès-Nancy, France; nviolle@etap-lab.com; 4 Ingredia, F-62000 Arras, France; boulier.audrey@gmail.com (A.B.); jessica.schwarz@outlook.com (J.S.); 5 Université de Lorraine, CALBINOTOX, F-54000 Nancy, France; zeeshan.hafeez@univ-lorraine.fr

**Keywords:** α-casozepine, anxiolysis, casein tryptic hydrolysate, GABAA, vagotomy

## Abstract

(1) Background: A tryptic hydrolysate of bovine α_s1_-casein (CH) exerts anxiolytic-like properties in many species, including humans. This is mainly related to the presence of α-casozepine (α-CZP), which yields these properties in rodents. This study evaluates, in a rat model, the roles of the vagus nerve and the benzodiazepine binding site of GABA_A_ receptors in the mode of action of CH. (2) Methods: The conditioned defensive burying test was used to evaluate anxiety. (3) Results: Participation of the vagus nerve in the mode of action of CH was excluded, as the global anxiety score in vagotomised rats was not significantly different from that of non-vagotomised animals. The blocking of the binding sites of benzodiazepines with flumazenil antagonised CH anxiolytic-like properties. (4) Conclusions: The vagus nerve does not play a role in the anxiolytic-like properties of CH. On the other hand, this anxiolytic-like activity relies on the benzodiazepine binding site of the GABA_A_ receptors. This result is consistent with previous in vitro studies and, more specifically with the discovery of α-CZP, the peptide responsible for the anxiolytic-like properties of CH.

## 1. Introduction

Hydrolysis of milk proteins, especially caseins, releases peptides, some of which display biological activity, as demonstrated in vitro, less frequently in vivo and rarely in clinical studies [1]. A tryptic hydrolysate of bovine αs1-casein exerts anxiolytic-like and anti-convulsant effects in rats [2], and the industrial product obtained after scale-up (CH, Lactium^®^) exhibits the same anxiolytic-like effects in rats [3] and in humans [4,5], as well as sleep-modulating properties in rats [6]. The anxiolytic-like properties of CH were confirmed amongst other species, such as cats [7], dogs [8], ponies [9], and horses [10]. The anxiolytic-like properties of hydrolysates were attributed to the presence of the tryptic fragment 91–100 of bovine α_s1_-casein (YLGYLEQLLR), called α-casozepine (α-CZP), which displays these effects in rats [2] and mice [11]. In vitro digestion of α-CZP yielded shorter N-terminal peptides, YLGYLEQ and YLGYL, which also possess anxiolytic-like properties in rats [12] and mice [13], respectively, and could then contribute to the in vivo property of α-CZP (for a general review on CH and α-CZP, see [14]).

Anxiety is a complex phenomenon involving several brain regions [15] and different neurotransmitter systems, including the GABAergic system, the target of benzodiazepines, the most prescribed anxiolytic drugs [16]. α-CZP exhibits affinity for the benzodiazepine site of GABA_A_ receptors and has been screened as a consequence of this affinity, but this one is 10,000 times lower than that of the benzodiazepine diazepam (Dzp) [2]. Furthermore, bicuculline, a GABA_A_ receptor antagonist, blocked the CH effect on chloride ion influx in neuroblastoma cell culture [17]. In spite of that, it is interesting to note that, unlike Dzp, CH did not induce the typical side effects of benzodiazepines, such as memory impairment, tolerance, or dependence, in rats [18].

The aim of this study is to better understand the mechanism by which CH displays its anxiolytic-like activities. A number of arguments support that the mode of action of the hydrolysate, especially of its active peptide α-CZP, should be central [11,13]. Nevertheless, the question of a peripheral mechanism of action in the intestine remains, particularly because CH also exhibits an anxiolytic-like activity after oral administration. Indeed, as shown recently, the gut–brain axis, especially the vagus nerve, plays a role in anxiety regulation. Vagal afferents modulate neurotransmitters in the key areas of the limbic system [19], and the anxiolytic-like effects of some orally administered probiotics are mediated by the vagus nerve [20,21]. Moreover, it has been shown that an undecapeptide from soy β-conglycinin displayed anxiolytic-like effects in mice through a brain–gut interaction [22]. Thus, in this present work, the first experiment assesses the activity of an orally administered single dose of CH after subdiaphragmatic vagotomy in rats. The second experiment assesses the implication of the benzodiazepine binding site of GABA_A_ receptors in the in vivo anxiolytic-like activities of CH using the antagonist flumazenil [23]. The anxiolytic-like properties of CH are evaluated using the conditioned burying test according to Pinel and Treit’s work [24], as the anxiolytic-like activities of the hydrolysate were well assessed in this procedure [2,3].

## 2. Materials and Methods

### 2.1. Animals

The experiment using the GABA_A_ receptor antagonist (Experiment 2) was approved by the French “Ministère de l’Agriculture, de l’Agroalimentaire et de la Forêt” on the recommendation of the “Comité d’Éthique Lorrain en Matière d’Expérimentation Animale” (Project number 6452). The assessment of the activity of orally administered CH after subdiaphragmatic vagotomy (Experiment 1) was in compliance with European Communities Council Directive 86/609/EEC (no longer in force). Experiment 1 was carried out using 24 male Wistar rats (HsdBrlHan, Harlan, Melderslo, The Netherlands), weighing 250–275 g at their arrival. Experiment 2 was carried out on 96 male Wistar rats (Charles River Laboratories, Saint-Germain-Nuelles, France), weighing 250–275 g at their arrival. Rats were group-housed, four per cage, in polycarbonate cages 48 × 27 × 20 cm (U.A.R., Epinay-Sur-Orge, France) in a regulated environment (humidity: 55 ± 20%; temperature: 22 ± 1 °C; lights off: 08:00 a.m.–08:00 p.m.). Rats were allowed free access to food (food pellets M20, Dietex, Argenteuil, France, for Experiment 1 or food pellets M20, Special Diets Service, Whitam, UK, for Experiment 2) and tap water until the day before the experiments. Food pellets M20 do not contain any dairy protein. After an acclimation period of 7 days after the day of their arrival, the rats were randomly assigned into three groups (n = 8/group) for Experiment 1 or six groups for Experiment 2 (n = 16/group). The rats in the different groups were all handled in the same way and under the same conditions.

### 2.2. Experimental Procedure

#### 2.2.1. Experiment 1: Vagus Nerve Involvement in Anxiolytic Activity of CH

Sixteen animals belonging to two of the three groups were anesthetised with an i.p. injection of 2 mg/kg acepromazine maleate (Calmivet, Vétoquinol, Lure, France), followed by an i.p. injection of 50 mg/kg ketamine (Virbac, Carros, France), and then subjected to a complete subdiaphragmatic vagotomy (n = 8) or sham operation (n = 8). Briefly, after laparotomy, the two trunks of the vagus nerve were identified under an operating microscope. Both trunks were cut off close to the diaphragm. For the sham vagotomy, the vagus nerve was similarly exposed but was not cut. After surgery, a recovery period of 3 weeks was allowed before the conditioned defensive burying test (described below). For this test, CH (Lactium^®^, Ingredia, Arras, France) was dissolved at 3 mg/mL in distilled water before an oral administration (orogastric gavage) to the sham-operated and vagotomised rats at a single dose of 15 mg/kg one hour before testing. Three days after the conditioned defensive burying test, the success of the vagotomies was verified by the measure of food intake after an i.p. injection of the neuropeptide CCK-8S (Sigma, Saint-Quentin-Fallavier, France) at a dose of 4 µg/kg.

#### 2.2.2. Experiment 2: Benzodiazepine Site of GABAA Receptor Involvement in Anxiolytic Activity of CH

CH (Lactium^®^, Ingredia, Arras, France) was dissolved at 3 mg/mL in distilled water. Diazepam (Roche, Meylan, France) was suspended at 0.6 mg/mL in a 0.4% (*v*/*v*) Tween 80 and 0.4% (*w*/*v*) methylcellulose aqueous solution. The solution used for the suspension of Dzp without the active molecule served as the vehicle for the oral administration of the control groups (vehicle 1). Flumazenil (Sigma-Aldrich, Saint-Quentin-Fallavier, France) was suspended at 2 mg/mL in a 0.9% NaCl and 2% (*v*/*v*) Tween 90 aqueous solution. The solution used for the suspension of flumazenil without the active molecule served as the vehicle for the i.p. injection for the control groups (vehicle 2). Groups are summed up in Table 1.

Injections by i.p. way of flumazenil or vehicle 2 (2 mL/kg) were performed 80 min before the test, while oral administrations of CH, diazepam, or vehicle 1 (5 mL/kg) were performed 60 min before the test.

The CH oral dose of 15 mg/kg was chosen because previous studies provided significant evidence of its efficiency in rats in both conditioned defensive burying and elevated plus-maze tests highlighting anxiolytic activity [3] and in the reduction of sleep disturbance induced by mild chronic stress [6].

### 2.3. Conditioned Defensive Burying Test

#### 2.3.1. Apparatus

The conditioned defensive burying test used in the present study is based on Pinel and Treit’s procedure [24]. Habituation, shocking and testing were done in a 45 × 28 × 18 cm clear Plexiglas chamber, the floor of which was evenly covered by 5 cm of bedding material made of wood sawdust. On the center of one wall, 2 cm above the level of the bedding material, a shock probe could be inserted via a small hole. The shock probe consisted of a 7 × 2 × 0.5 cm Plexiglas slide overlaid with a copper-wire-integrated circuit connected to an electric shock generator, which can deliver 0 to 8 mA. The operator manually handled the release of the electric shock. In the slightly lit test room, a CCD-TV camera allowed the rats to be observed and recorded from a neighbouring room.

#### 2.3.2. Procedure

For habituation to the experimental conditions, each home cage group was placed in the test chamber without the shock probe for 20 min during the two days prior to the test. The shock probe was inserted into the chamber before the test session. Rats were individually placed in the test chamber, on the side opposite the shock probe. The first time the rat touched the probe with its forepaws, the experimenter delivered a single 2 mA shock. Immediately after shock administration, the behaviour of each rat was recorded for 5 min. Bedding material was changed each day of testing and smoothed to a uniform depth of 5 cm between each rat test. Rats that did not touch the probe within 5 min were excluded from the study. The conditioned defensive burying tests were performed during the first 3 h of the dark cycle, the period when the rats are more active.

#### 2.3.3. Studied Parameters

The following parameters were scored by an experimenter unaware of the group setup: duration of probe-burying, number of head stretchings towards the probe, number of approaches towards the probe and number of retreats away from the probe. The “number of approaches” and the “number of retreats” allowed us to calculate the “percentage of approaches towards the probe followed by retreats”:

(1) $$\frac{\mathrm{number}\;\mathrm{of}\;\mathrm{retreats}}{\mathrm{number}\;\mathrm{of}\;\mathrm{approaches}}\times 100$$

Within each of the variables of “duration of probe-burying”, “number of head stretchings towards the probe” and “percentage of approaches towards the probe followed by retreats”, all values were classified in increasing order and then transformed in their respective ranks. For each rat, the sum of the ranks of the three variables represents its global score of anxiety.

### 2.4. Statistical Analysis

All data are expressed in percentage to the vehicle group (100%) and are reported as mean ± SEM. Data were evaluated with a Kruskall–Wallis test using R [25]. To further show two-by-two group differences, a Mann–Whitney U-test was performed for each experimental group in comparison with the control group of each experiment. Differences were considered to be significant at *p* < 0.05.

## 3. Results

### 3.1. Evaluation of Anxiolytic Properties of CH after Oral Administration in Vagotomised Rats

**Figure 1:**
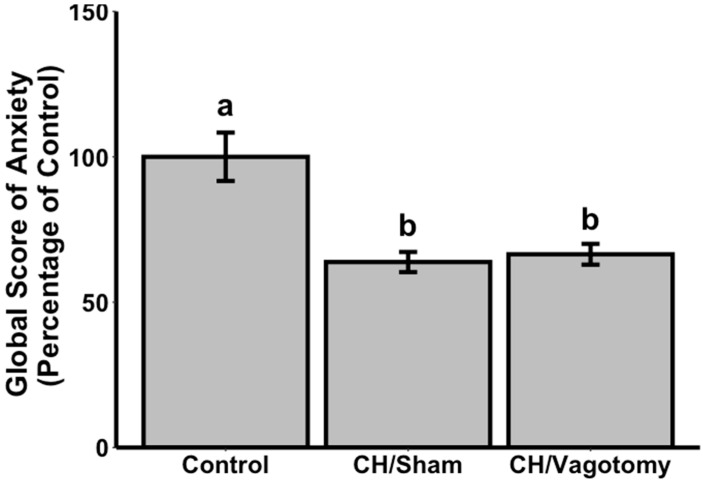
Effects of CH, orally administered at a single dose of 15 mg/kg, on the global score of anxiety in a conditioned defensive burying model in vagotomised male Wistar rats (n = 8 / group). Data are mean ± SEM. Means with different letters are significantly different (Mann–Whitney U-test, *p* < 0.05). CH, tryptic hydrolysate of bovine α_s1_-casein.

There was a significant effect of the combination of treatment and surgery on the global score of anxiety (H_(2)_ = 14.432). Compared to vehicle, CH orally administered at a single dose of 15 mg/kg significantly decreased the global score of anxiety in the conditioned defensive burying test of sham male Wistar rats (−34%, U = 54, *p* = 0.003; Figure 1). A significant decrease in the global score of anxiety compared to the vehicle group was also observed for the vagotomised rats (−36%, U = 62, *p* = 0.001). No difference between sham-operated and vagotomised rats receiving CH was detected (+4%, U = 23, *p* = 0.535). Surgery was a success for all vagotomised animals as it was shown that food intake did not decrease after an i.p. injection of CCK-8S (data not shown), the CCK satiating action being mediated by the vagus nerve [26].

### 3.2. Effects of Flumazenil on Anxiolytic Properties of Orally Administrated CH

**Figure 2:**
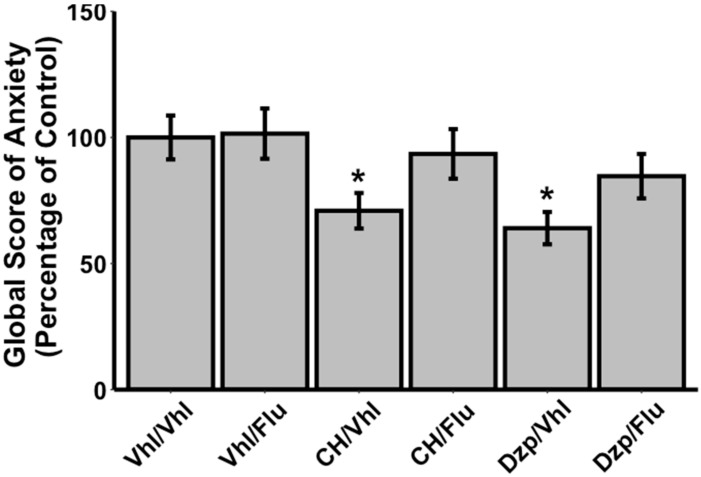
Effects of flumazenil (10 mg/kg, i.p.) on CH (15 mg/kg, oral) anxiolytic action in a conditioned defensive burying model in rats (n = 11–15 / group). Data are mean ± SEM. * Indicates significant differences between the experimental group and the Vhl/Vhl group (Mann–Whitney U-test, *p* < 0.05). Vhl, vehicle; Flu, flumazenil; CH, tryptic hydrolysate of bovine α_s1_-casein; Dzp, diazepam.

There was a significant effect of the combination of treatments on the global score of anxiety (H_(5)_ = 13.599, *p* = 0.02). Animals in both the CH/Vhl and Dzp/Vhl groups showed a significantly lower global score of anxiety in comparison to rats in the Vhl/Vhl group (−29%, U = 103, *p* = 0.024 and −36%, U = 130.5, *p* = 0.004, respectively; Figure 2). As far as the effects of the antagonist are concerned, flumazenil had no effect on the global score of anxiety, as there was no significant difference between the Vhl/Vhl and Vhl/Flu groups (+2%, U = 92, *p* = 0.942). The flumazenil injection reversed the effects of diazepam when administered 20 min before this drug, as the global score of anxiety increased when the Dzp/Flu group was compared to the Dzp/Vhl group (+32%), and no difference was observed between the Vhl/Vhl and Dzp/Flu groups (−15%, U = 98, *p* = 0.329). Concerning the CH-treated groups, a flumazenil i.p. injection 20 min before the administration of CH (CH/Flu) increased the global score of anxiety compared to vehicle injection (CH/Vhl; +32%), and no difference was observed between the Vhl/Vhl and CH/Flu groups (−7%, U = 91, *p* = 0.496).

## 4. Discussion

The tryptic hydrolysate of bovine α_s1_-casein containing α-CZP has displayed anxiolytic-like properties in numerous species, including human beings [14]. Our results showed that the anxiolytic-like action of CH is independent of the vagus nerve. The mode of action of CH involves the benzodiazepine site of the GABA_A_ receptor, as its anxiolytic-like properties were reversed in vivo by flumazenil, which has a high affinity for the benzodiazepine site of GABA_A_ receptors.

CH may have a central mechanism of action as it displayed anxiolytic-like activity in vivo after i.p. administration [2]. This was also the case for α-CZP [2] and the peptides 91–95 [13] and 91–97 [12] generated after its proteolysis. Vagal afferents connect the brain via the nucleus of the tractus solitarius (NTS). A previous study showed that neither α-CZP nor peptide 91–95 significantly increased c-Fos protein expression in the NTS after i.p. administration [13], but c-Fos expression was not measured after oral administration. Hence, exclusion of the vagus nerve from the mechanism of action of CH, which contains α-CZP, had to be demonstrated. Indeed, it was shown that some peptides derived from the hydrolysis of dietary proteins can exert their biological effect via the vagus nerve but are also active after i.p. administration. Thus, soy-deprestatin displayed an antidepressant-like activity only after oral administration, activity which was mediated by the vagus nerve [27]. In contrast, the undecapeptide derived from the α-subunit of soy β-conglycinin, βCGα (323–333), exerted anxiolytic-like activity after 1 mg/kg oral or i.p. administration and the activity after per os administration was abolished after vagotomy [22]. Several types of mechanisms that are not totally elucidated concerning the action of biologically active peptides from food proteins may exist.

Vagotomy in rats, orally administered with CH, did not suppress the effect of this hydrolysate on the global score of anxiety in the conditioned defensive burying test. The anxiolytic-like activity of this hydrolysate in these animals was similar to that observed in sham-operated rats. Thus, the anxiolytic-like properties of CH are not mediated by the vagus nerve, meaning that the peptide(s) carrying the bioactivity inside CH may reach its (their) pharmacological target(s) at a level that should not be the intestinal one. Of the different peptides that compose the CH, it has been shown that α-CZP, tryptic fragment 91–100 of the bovine α_s1_-casein, is a carrier of CH anxiolytic-like activity [2]. In vitro digestibility of this peptide has already been assessed and the fragment corresponding to the sequence 91–97 was found in significant amounts in the medium, especially after hydrolysis either by pepsin or by pepsin followed by Corolase^®^ PP, and was shown to possess comparable anxiolytic-like properties as α-CZP [12]. Moreover, it has been stated that the peptide bonds in the N-terminal region (between residues 91 to 95) of α-CZP are notably resistant to different proteases (pepsin, chymotrypsin, and Corolase^®^ PP). Peptide 91–95 was also recovered in significant amounts in the medium after the in vitro digestion of α-CZP either by Corolase^®^ PP or by pepsin followed by Corolase^®^ PP [12]. This shorter peptide also displays anxiolytic-like properties in mice [13]. Finally, it has been reported that α-CZP transport across Caco-2 cells is facilitated in the presence of bile salts and that these conditions lead to a higher formation of the 91–97 fragment by the proteolytic machinery of this cell line [28]. These results agree with an action, after oral administration, of one or more peptides coming from the CH and/or from the digestion of one or more peptides contained in it, such as α-CZP, via the bloodstream. The peptides 91–97 and 91–95 could be candidates as carriers of this anxiolytic activity. In vitro simulated gastrointestinal digestion of a whole casein tryptic hydrolysate containing α-CZP showed that peptides 91–95 and 91–97 were present at a significant level after 120 min of digestion, peptide 91–97 having even the higher concentration among the fragments coming from α-CZP after 180 and 240 min [29]. Nevertheless, the mode of action of all these anxiolytic peptides might be slightly different, as was observed when comparing the modulation of the neuronal activity of the amygdala by α-CZP and peptide 91–95 YLGYL [13].

The mode of action of CH is reliant on the benzodiazepine site, which is a specific binding site of these molecules on GABA_A_ receptors, allowing them to act as positive allosteric modulators on these receptors [30]. This result is coherent with previous in vitro studies as α-CZP, the bioactive peptide displaying the same anxiolytic-like properties as CH, was screened over this specific binding site by displacing [methyl-^3^H]-flunitrazepam from the benzodiazepine site on GABA_A_ receptors [2]. A more recent study showed that α-CZP had a chEMBL score of more than 83 in a docking study with the benzodiazepine site of the GABA_A_ receptor compared to a score of about 78 for alprazolam, a sedative benzodiazepine [29]. Moreover, bicuculline, an antagonist that binds in the orthosteric site of GABA_A_ receptors [31], blocked the effects of CH on Cl^–^ influx in a neuroblastoma cell culture, also indicating a role of GABA_A_ receptors in the mode of action of CH [17]. GABA_A_ receptors are composed of 5 subunits and show at least 19 different combinations of subunits associated with different localisations in the brain as well as different properties. The α_2_ subunit seems to be implicated in the anxiolytic properties of these receptors [32]. The apparent affinity of α-CZP in competition with tritiated flunitrazepam for the benzodiazepine site of GABA_A_ receptors is 10,000 times lower than the affinity of diazepam in the same conditions [2]. Flunitrazepam binds to GABA_A_ receptors composed of α_1_, α_2_, α_3_ or α_5_ subunits, in addition to β and γ subunits, and an affinity was even highlighted on receptors possessing an α_4_ subunit associated with the β_3_ subunit [33]. Flunitrazepam is a non-selective benzodiazepine, and, therefore, more than 90% of GABA_A_ receptors of the central nervous system are targeted by it. As the tryptic hydrolysate does not have the side effects of benzodiazepines, α-CZP, or a shorter derived peptide such as YLGYL (peptide 91–95) or YLGYLEQ (peptide 91–97), might bind only to a specific population of GABA_A_ receptor subtypes; this could explain the lower affinity of this peptide for the global population of GABA_A_ receptors that was determined by competition with [methyl-^3^H]-flunitrazepam. This means that the population of GABA_A_ receptors targeted by the peptide(s) might be narrower than that targeted by flunitrazepam. Further experiments using specific GABA_A_ subtype antagonists could be interesting to carry on with.

Previous work on anxiolytic peptides derived from bovine α_s1_-casein showed that after an i.p. administration of α-CZP in mice, the anxiolytic-like properties of this peptide (in an elevated plus-maze model) were blocked by the inhibition of 5-HT_1A_, dopamine D_1_, or GABA_A_ receptors by WAY100135, SCH-23390, and bicuculline respectively [34]. A smaller peptide, YL, which corresponds to fragments 91–92 and 94–95 of bovine α_s1_-casein but may also be released from numerous proteins since this sequence frequency is about 0.28% [35], also possesses anxiolytic-like properties, which are mediated by 5-HT_1A_, dopamine D_1_, and GABA_A_ receptors despite having no affinity for any of these receptors [36]. This dipeptide could trigger the different receptors in the following order: 5-HT_1A_, dopamine D_1_, and, eventually, the GABA_A_ receptor. Dipeptide YL was not found after in vitro digestion of α-CZP by pepsin and/or Corolase^®^ PP [12] and was present at only 0.03% after the digestion of bovine α_s1_-casein [34]. The observed differences between the results obtained with the anxiolytic peptides derived from bovine α_s1_-casein should implicate different modes of action [13]. A specific binding of α-CZP on GABA_A_ receptor subtypes that are involved in anxiolysis (α_2_-containing GABA_A_ receptors [37]) but not to those involved in amnesia, addiction or sedation (α_1_-containing GABA_A_ receptors [38]) could explain the absence of side effects for CH, which are traditionally associated with benzodiazepines (memory impairment, tolerance or dependence) [18]. Other peptides present in CH could also interfere with α-CZP and modulate its anxiolytic-like properties, thus also explaining the slight differences observed between the active doses of CH and α-CZP since the hydrolysate containing α-CZP is more effective than the corresponding dose of pure α-CZP.

Our results strongly reinforce the previous hypotheses suggesting that the mode of action of CH was central. Indeed, CH, which contains α-CZP, exhibited an anxiolytic-like activity after oral administration without involving the vagus nerve. Moreover, the mechanism of action implied the benzodiazepine site of the GABA_A_ receptor since the administration of flumazenil antagonised CH activity. Flumazenil, similar to benzodiazepines and Z-drugs, binds in a pocket under the C-loop of the GABA_A_ receptor at the interface of the α and γ subunits [39]. Flumazenil binds less deeply in the pocket than diazepam, indicating that common structural patterns do not bind in the same way. These differences could also explain selectivity towards GABA_A_ receptor subtypes and differences in the activity of molecules [39]. This observation might apply to the active peptide(s) derived from CH. How could a hydrolysate (administered per os) act on the benzodiazepine site of the GABA_A_ receptor, which is an extracellular site belonging to a receptor that is synaptic, perisynaptic or extrasynaptic? α-CZP is active after i.p. injection but can also undergo digestive proteolysis to generate shorter fragments. YLGYL, YLGYLEQ or YL also display anxiolytic-like activity after i.p. injection [12,13,36]. It could be suggested that only shorter peptides could be absorbed in the intestine, and the number of potential active peptides at a central level could, hence, be reduced. Nevertheless, stress has been shown to increase the permeability of the small intestine in humans [40], and the action of α-CZP was only highlighted in stressed animals [11]. Moreover, the presence of bile salts in an intestinal absorption model increases the transfer of α-CZP and YLGYLEQ [28]. Actions of the longer peptides cannot, therefore, be excluded because α-CZP and YLGYL modulate (after an i.p. injection) the expression of c-Fos in different brain structures implicated in the regulation of anxiety [11,13]. After the absorption step, the active peptide(s) must then reach the brain and cross the blood–brain barrier. This step is currently a black box and would require experiments. The low affinity of α-CZP for the benzodiazepine site of GABA_A_ receptors does not exclude this peptide from being one of the active molecules since the efficacy in modulating GABA_A_ receptor subtypes is the more important parameter to understand the activity of a drug compared to its affinity [41]. Nevertheless, the understanding of the mechanism in the brain is complicated by the fact that the peptides YL, YLGYL or YLGYLEQ exhibit an anxiolytic action, which does not necessarily involve the same pathways and which, in the case of YLGYL, does not modulate the expression of c-Fos in the same way as α-CZP in the amygdala [13]. Although the present work has improved the understanding of the mechanism of action of CH administered orally, this mechanism remains complex and will require further studies to understand the properties of the different peptides. Another hypothesis would be that the action on the benzodiazepine site of the GABA_A_ receptor might not be directly due to peptide(s) coming from CH but might be indirect, involving endozepines. Indeed, these molecules might positively or negatively modulate the GABA_A_ receptor via the benzodiazepine site, and flumazenil seems to be able to inhibit the action of these endogenous molecules on this site [41]. However, these findings regarding endozepines and the benzodiazepine site of the GABA_A_ receptor still need to be reinforced.

## Figures and Tables

**Table 1:** Summary of the tested products, used doses, ways of administration, time of administration before testing and names of experimental groups analyzing the involvement of the benzodiazepine site of GABA_A_ receptors in the anxiolytic activity of CH. Rats that did not touch the probe were removed from the experiment (final n compared to initial n). Vehicle 1 corresponds to the solution for the suspension of diazepam and vehicle 2 to that for the suspension of flumazenil.

	Antagonist		Test Product		Initial n	Final n
Group Name	Dose, Administration	Time before Test	Dose, Administration	Time before Test		
Vhl/Vhl	Vehicle 2 (2 mL/kg, i.p.)	80 min	Vehicle 1 (5 mL/kg, p.o.)	60 min	16	12
Vhl/Flu	Flumazenil (10 mg/kg, i.p.)	80 min	Vehicle 1 (5 mL/kg, p.o.)	60 min	16	15
CH/Vhl	Vehicle 2 (2 mL/kg, i.p.)	80 min	CH (15 mg/kg, p.o.)	60 min	16	11
CH/Flu	Flumazenil (10 mg/kg, i.p.)	80 min	CH (15 mg/kg, p.o.)	60 min	16	13
Dzp/Vhl	Vehicle 2 (2 mL/kg, i.p.)	80 min	Diazepam (3 mg/kg, p.o.)	60 min	16	13
Dzp/Flu	Flumazenil (10 mg/kg, i.p.)	80 min	Diazepam (3 mg/kg, p.o.)	60 min	16	13

Vhl, vehicle; Flu, flumazenil; CH, tryptic hydrolysate of bovine αs1-casein; Dzp, diazepam.

## Data Availability

Not applicable.
